# Chitosan Oligosaccharides Mitigate Flooding Stress Damage in Rice by Affecting Antioxidants, Osmoregulation, and Hormones

**DOI:** 10.3390/antiox13050521

**Published:** 2024-04-26

**Authors:** Haoyu Lu, Mei Wang, Shangfeng Zhou, Ke Chen, Lifeng Wang, Zhenxie Yi, Lianyang Bai, Yuzhu Zhang

**Affiliations:** 1 State Key Laboratory of Hybrid Rice, Hunan Hybrid Rice Research Center, Changsha 410125, China; 13319501999@163.com (H.L.); 15574808897@163.com (M.W.); answer514367672@163.com (K.C.); ifwang@hunaas.cn (L.W.); 2 College of Agronomy, Hunan Agricultural University, Changsha 410128, China; yizhenxie@126.com; 3 Hunan Agricultural Biotechnology Research Institute, Changsha 410125, China; sfzhou7389@hunaas.cn

**Keywords:** rice, flooding stress, chitosan oligosaccharide, antioxidant system, hormones

## Abstract

Rice (*Oryza sativa* L.) is one of the most important food crops worldwide. However, during direct seeding, rice is extremely vulnerable to flooding stress, which impairs rice’s emergence and seedling growth and results in a significant yield loss. According to our research, chitosan oligosaccharides have the potential to be a chemical seed-soaking agent that greatly increases rice’s resistance to flooding. Chitosan oligosaccharides were able to enhance seed energy supply, osmoregulation, and antioxidant capacity, according to physiological index assessments. Using transcriptome and metabolomic analysis, we discovered that important differential metabolites and genes were involved in the signaling pathway for hormone synthesis and antioxidant capacity. Exogenous chitosan oligosaccharides specifically and significantly inhibit genes linked to auxin, jasmonic acid, and abscisic acid. This suggested that applying chitosan oligosaccharides could stabilize seedling growth and development by controlling associated hormones and reducing flooding stress by enhancing membrane stability and antioxidant capacity. Finally, we verified the effectiveness of exogenous chitosan oligosaccharides imbibed in seeds by field validation, demonstrating that they can enhance rice seedling emergence and growth under flooding stress.

## 1. Introduction

Currently, global food security is being greatly challenged by climate change, and rice, being a primary food crop in Asia, plays a significant role in addressing this issue [1]. With the increase in agricultural production costs, labor scarcity, and the increase in agricultural large-scale management areas, the direct seeding mode of rice has been gradually replacing the transplanting mode. In the process of the direct seeding mode, rice seeds will be directly sown in the soil without nursery seedlings, so the seeds will face a variety of abiotic stresses such as cold damage, drought, and flooding in the germination and seedling growth stages [2,3]. Flooding is one of the most common abiotic stresses in the process of directly seeding rice. No matter whether dry direct seeding, wet direct seeding, or water direct seeding is used, it is difficult to avoid the occurrence of flooding stress. The main reason is that during the early stage of the direct seeding of rice, unpredictable strong monsoon rainfall, poor land leveling, and poor drainage cause flooding stress, which affects the germination rate, seedling emergence rate, root length, and fresh weight of rice, and finally severely affects the yield [4].

In the case of flooding stress, partial or complete immersion of rice in water will limit the diffusion of oxygen to plants and, thus, inhibit their aerobic respiration [5,6]. During the process of flooding stress, rice seeds and seedlings will undergo a series of physiological and morphological changes, such as the imbalance of osmotic potential, accelerated oxidative damage, coleoptile elongation, and inhibition of root growth [7,8,9,10]. Rice, as the only food crop that can germinate and grow seedlings under completely flooded conditions, has evolved a variety of physiological and morphological mechanisms to adapt to flooding stress, including the formation of aerenchyma; gibberellin and ethylene promote internode elongation to allow plants to extend above the water surface for gas exchange (an adaptation of deep-water rice); and inhibition of growth and conservation energy until the flood recedes (an adaptation of lowland rice) [11,12,13,14]. These adaptations enable rice to survive and reproduce in different ecological environments.

Seed soaking with chemicals has long been regarded as an effective technology to improve seed germination and resist environmental stress [15,16,17]. Chemical soaking provides seeds with physiological, molecular, and metabolic changes and high tolerance to different abiotic stresses during germination [18]. At present, a variety of chemicals have been proven to be able to soak seeds and improve the stress resistance during seed germination and seedling growth. For example, spermidine and 5-aminolevulinic acid can alleviate cold stress, salicylic acid and polyethylene glycol (PEG) can alleviate drought stress, and chitosan and melatonin can alleviate salt stress [19,20,21,22,23].

Chitosan is a linear polysaccharide similar in structure to cellulose and is abundant in nature. Chitosan oligosaccharides can be obtained by physical, chemical, or biological enzymatic degradation of chitosan with a molecular weight of <4 kDa [24]. Chitosan oligosaccharide, as a natural product with good water solubility, large functionality, and high biological activity, has a wide range of applications in plant growth and development involving many plant species [25]. Although the mechanism of action of chitosan oligosaccharides in plants has not been fully investigated, studies have reported it to be related to the activation of the plant defense system, promotion of plant growth and development, regulation of phytohormone levels, and enhancement of plant antioxidant capacity [24,26,27,28].

At present, many studies on the resistance of chitosan oligosaccharides to different plant stresses have been published [29]. But the research on the resistance of rice to flooding stress has not been reported. This study determines the physiological characteristics of the effects of chitosan oligosaccharide pretreatment on rice’s resistance to flooding stress through physiological measurement and multi-omics sequencing, explores its molecular mechanism, and verifies its effectiveness in field experiments. This research suggests that chitosan oligosaccharides can resist the damage caused by flooding stress by inhibiting the production of reactive oxygen species and affecting the anabolism of tryptophan, auxin, jasmonic acid, and abscisic acid.

## 2. Materials and Methods

### 2.1. Plant Cultivation and Treatments

Rice seeds were sterilized with 1.5% (*v*/*v*) sodium hypochlorite for 20 min and rinsed with double-distilled water (ddH_2_O). Chitosan oligosaccharide treatment (COS): soaked in 50 mg/L chitosan oligosaccharides at 26 °C for 40 h. Flooding treatment (AG): soaked in water at 26 °C for 40 h. Moistened and germinated at 36 °C for 12 h until seeds germinated and reached about 1 mm in length. Rice seeds that met the criteria were placed within the bottom open cells of PCR plates (with an opening radius of 1.2 mm). The seeded PCR plates were placed on a light-shielding box inside the apparatus, to which a cover was subsequently added. After that, those were placed inside an artificial climate chamber (FH-1200, TAIWAN HIPOINT, Kaohsiung, China). The parameters of the artificial climate chamber were as follows: temperature was set to 26 degrees Celsius, 50% humidity, 14 h of light with the light intensity of 5000 lx and 10 h of darkness. Both treatments were submerged with water level of 9 cm, and after the submergence, stress was maintained for 9 days. Two blank controls (CK and CK+COS) were seeded on the same PCR plate after using water-soaked and chitosan oligosaccharide-soaked, respectively, and then placed in an immersion identification device. The seeds were submerged in water to a depth of half their size. The treatment of the different chemicals and concentrations was consistent with the previous section, adjusted only for their chemical nature and concentration, with the specific concentrations of the different substances shown in Table S2. The rice seeds involved in this study were all provided by Hunan Golden Nongfeng Seed Industry Co., Ltd. (Changsha, China). Chitosan oligosaccharides were purchased from Tokyo Chemical Industry Co., Ltd. (Tokyo, Japan). The degree of polymerization of chitosan oligosaccharides is 4–5.

The height of the seedlings was measured daily using a vernier caliper. The root length was measured using a vernier caliper 8 days after sowing. Fresh weight was weighed after using a tissue to absorb surface water after sampling. Seedlings and seeds treated with AG and COS were taken 6 days after sowing, frozen in liquid nitrogen, and stored at −80 °C for further study.

### 2.2. Biochemical Assays

Relative conductivity (EC) was measured with the METTLER TOLEDO Conductivity Meter (FE30, METTLER TOLEDO, Shanghai, China). Three seedlings were rinsed with distilled water, placed in 10 mL of distilled water, immersed for 4 h, and shaken to measure the initial electronic conductance (S1). The samples were boiled in a water bath for 10 min and the final conductance (S2) was measured after cooling to room temperature. Electrical conductivity was evaluated as EC (%) = S1/S2 [30]. Biochemical index determination kits (Abbkine Scientific Co., Ltd., Wuhan, China) were used for the determination of superoxide dismutase activity (SOD), catalase activity (CAT), glutathione s-transferase activity (GST), α-amylase activity, malondialdehyde content (MDA), proline content (Pro), soluble protein content, and soluble sugar content [31].

### 2.3. RNA Extraction, Library Construction, RNA Sequencing, and Data Analysis

Shanghai Ouyi Biotechnology Co. (Shanghai, China) used the Illumina sequencing platform to sequence and analyze transcriptomes. To guarantee dependable outcomes from the data, low-quality degrees (Q ≤ 20) were eliminated. After that, TopHat 2 (V2.1.1) assembled and mapped the data to the *Oryza sativa* L. genome. The predicted number of fragments per kilobase of transcript per million mapped fragments (FPKM) was used to express gene reads. Genes that were differentially expressed had to have an FPKM value of at least 10 and a false discovery rate (FDR) of at least 0.05. Kyoto Encyclopedia of the Genome (KEGG) and Gene Ontology (GO) enrichment were used for functional analysis [32].

### 2.4. Metabolite Profiling and Data Analysis

The same material used for transcriptome sequencing was used for metabolome sequencing, with six replicates per treatment. Shanghai Lumine Biotechnology Co. carried out the metabolite extraction and liquid chromatography–tandem mass spectrometry (LC-MS/MS) analysis. The metabolic profiles in ESI-positive and ESI-negative ion modes were analyzed using an AB SCIEX Triple TOF 5600 system (AB SCIEX, Framingham, MA, USA) and an ACQUITY UHPLC system (Waters Corporation, Milford, MA, USA). For both positive and negative ion modes (1.7 µm and 2.1 × 100 mm), an ACQUITY UPLC BEH C18 column was utilized. Progenesis QI data processing software (Waters Corporation, Milford, MA, USA) was used to process the metabolite data, and the identified metabolites were analyzed using custom databases for metabolite identification along with public databases (e.g., http://www.lipidmaps.org/, accessed on 1 April 2019) and http://www.hmdb.ca/ (accessed on 1 April 2019) that included public databases for metabolite identification. The criteria used to establish differential metabolites were VIP value > 1 and *p*-value < 0.05 [33].

### 2.5. Field Trial Verification

A split zone design was adopted, with the main zone being the rice variety and the subzone being the treatment method. Each treatment had four biological replicates, with ridges used to separate and irrigate each community separately (Figure S1). The test site was located in Huangpu District, Guangzhou (LON 113.23 E; LAT 23.06 N). The seeds were sown on 10 March 2023, and the meteorological data 30 days after sowing are shown in the schedule table (Table S1). The sowing rate was 3.75 g/m^2^. Blank treatment (CK): the seeds were soaked in water for 36 h, followed by 12 h of germination. The seeds were directly seeded onto the surface of the soil to keep the soil moist. Shallow water irrigation (water layer 2 ± 1 cm) was conducted 5 days after sowing, followed by alternating dry and wet irrigation. Flooding treatment (AG): the pre-sowing treatment was consistent with CK, and after 48 h of sowing, the water layer was maintained at 7 ± 1 cm until 30 days after sowing. Chitosan oligosaccharide treatment (COS): the seeds were soaked in a 50 mg/L chitosan oligosaccharide solution for 36 h, then rinsed, and germination was promoted for 12 h. The seeds were directly seeded on the surface of the soil, keeping the soil moist. After 48 h of sowing, the water layer was submerged to 7 ± 1 cm and maintained until 30 days after sowing. The rice seeds were provided by Hunan Golden Nongfeng Seed Industry Co., Ltd. (Changsha, China). Twenty seedlings were sampled from each plot 7 days after sowing, and the seedling height, root length, number of roots, and stem base width were measured using a vernier caliper. Fresh weight was weighed after using a tissue to absorb surface water after sampling. A 0.25-square-meter (50 × 50 cm) plastic frame was used to randomly select cells, and this was repeated five times. The number of rice seedlings in this area was calculated to calculate the basic seedlings per square meter.

### 2.6. Data Analysis

In this study, SPSS 20.0 (SPSS Inc., Chicago, IL, USA) statistical software was used to perform one-way analyses of variance (ANOVA), Duncan’s multiple comparisons, Pearson correlation analyses, principal component analyses, and stepwise regression analyses on this data contained three biological replications. For other data processing, Microsoft Excel 2019 software was used. Picture drawing was performed using https://www.chiplot.online/ (accessed on 15 August 2023) and Graphpad-Prism 8 (Graph PadPrism^TM^ Software Inc., San Diego, CA, USA).

## 3. Results

### 3.1. Seed Soaking with Chitosan Oligosaccharides Can Alleviate the Inhibitory Effect of Flooding Stress

In order to solve the flooding stress problem of rice production in direct seeding mode, chemical pretreatment was implemented to improve the submergence tolerance of rice seedlings. In the previous exploration, 10 chemicals were screened for research (Table S2). It was discovered that chitosan oligosaccharides can significantly alleviate the inhibition of growth due to flooding stress of rice seedlings, which is mainly reflected in the promotion of seedling length and root length. Subsequently, the indica rice variety “Zhongzao 39” (ZZ39), which is commonly used in Chinese rice production, was chosen as a follow-up study variety due to its poor submergence tolerance; it was identified as a rice variety with poor submergence tolerance in previous studies [5]. ZZ39 and the male parent variety “Huazhan” (HZ), which is commonly used in hybrid rice, were applied to verify the efficacy of different concentrations of chitosan oligosaccharides (Figure S2, Table S2). The result indicated that seed soaking with 50–1000 mg/L chitosan oligosaccharides could improve the seedlings’ length 4 days after flooding. Although seed soaking with 10 mg/L chitosan oligosaccharides could improve seedling length, the effect was worse than that of 50–1000 mg/L. From the growth dynamics, it can be found that the coleoptile grew rapidly in the first two days after the germinated seeds were subjected to flooding stress, and the seedlings of AG and COS were significantly taller than those of the blank control (CK). With the development of growth, the seedling lengths of the CK were significantly greater than those of the flooding treatment 4 days after sowing. At this time, the rapid elongation of the coleoptile sheath under flooding stress was unable to produce a height advantage. After the fourth day, seedling height was significantly higher in chitosan-treated seedlings.

**Figure 1:**
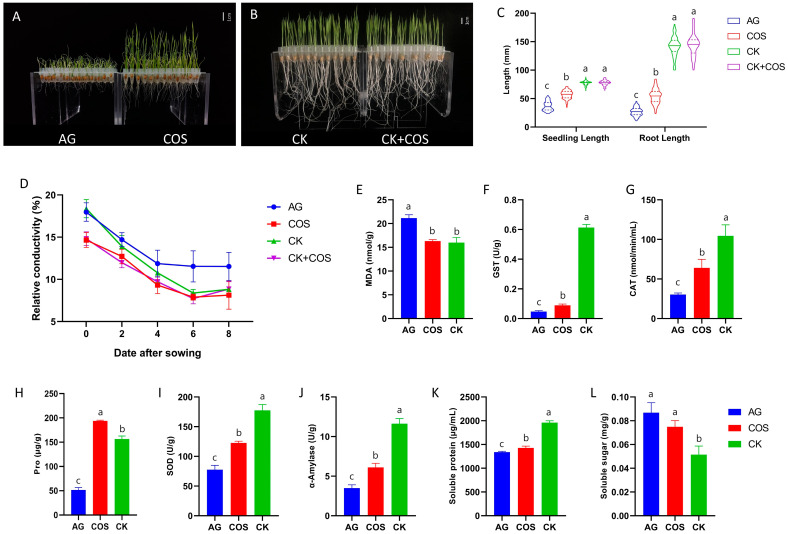
Phenotypic and physiological characterization of rice growth under flooding stress alleviated by chitosan oligosaccharides. (**A**,**B**) Phenotypic image. (**C**) Seedling length and root length. (**D**) Relative conductivity after seeding. (**E**) MDA content. (**F**) GST enzyme activity. (**G**) CAT enzyme activity. (**H**) Pro content. (**I**) SOD enzyme activity. (**J**) α-Amylase activity. (**K**) Soluble protein content. (**L**) Soluble sugar content. Columns with the same letter are not significantly different at *p* < 0.05, one-way ANOVA, followed by Tukey’s honestly significantly different tests.

According to the results of the concentration gradient test, after considering the practical application effect and cost in the field, 50 mg/L of the concentration was selected for the following studies. From the phenotype, it can be observed that seed soaking with chitosan oligosaccharides significantly alleviated flooding stress (Figure 1A,B). Compared to the CK group, the seedling height of the COS and AG treatments decreased by 26.57% and 52.34%, respectively. And the root length decreased by 61.80% and 81.00%, respectively. However, there was no significant difference in seedling height or root length between the CK and CK+COS treatments (Figure 1C, Table S2). Meanwhile, the dynamic relative electrical conductivity (EC) of the seedlings was measured (Figure 1D). The result was that EC decreased with sowing and tended to be stable 6–8 days after sowing. On d 0, the EC of the chitosan oligosaccharide pretreatment was significantly lower than that of the water-soaked seed. Under non-flooding stress, the EC of the CK+COS treatment was significantly lower than that of the CK at 0–2 days after sowing, and then there was no significant difference between them. Under flooding stress, the EC of the COS treatment was lower than that of the AG treatment at 0–8 days after sowing, and the reduction range was 31.61–13.54%. The above indicated that soaking seeds with chitosan oligosaccharides can greatly improve the tolerance of rice seedlings to flooding stress without affecting the normal growth of rice.

### 3.2. Physiological Characteristics of Seed Soaking with Chitosan Oligosaccharides to Improve the Flooding Tolerance of Rice

To further investigate the effect of soaking seeds in chitosan oligosaccharides on rice, seedlings from the flooding treatment (AG), the chitosan oligosaccharides soaking (COS) treatment, and the blank control (CK) were selected and tested. The physiological indices of seedings and seeds were measured 6 days after sowing (Figure 1E–L). MDA is a commonly used indicator in physiological studies of plant senescence and resistance, and the study found that the MDA content of AG was significantly higher than that of COS and CK treatments by 22.83% and 24.44%, respectively. Measurement of the antioxidant system revealed that SOD enzyme activity, CAT enzyme activity, and GST enzyme activity in AG were significantly lower than those of COS and CK treatments. Regarding proline (Pro) content, COS had the highest proline content, which was 73.35% and 19.43% higher than AG and CK treatments. At the same time, the study measured physiological indices related to osmoregulation and energy supply in seeds, and the results showed that soluble proteins were significantly lower in AG than in COS and CK treatments, whereas soluble sugars were significantly higher in AG than in COS and CK treatments. When α-amylase was measured, the α-amylase activity of AG was significantly lower than that of COS and CK. These results revealed that chitosan oligosaccharides had a significant effect on the antioxidant capacity, osmoregulation, and capacity supply of seeds after soaking.

### 3.3. RNA-Seq Analysis of Effect of Seed Soaking with Chitosan Oligosaccharides on Improving Rice’s Submergence Tolerance

**Figure 2:**
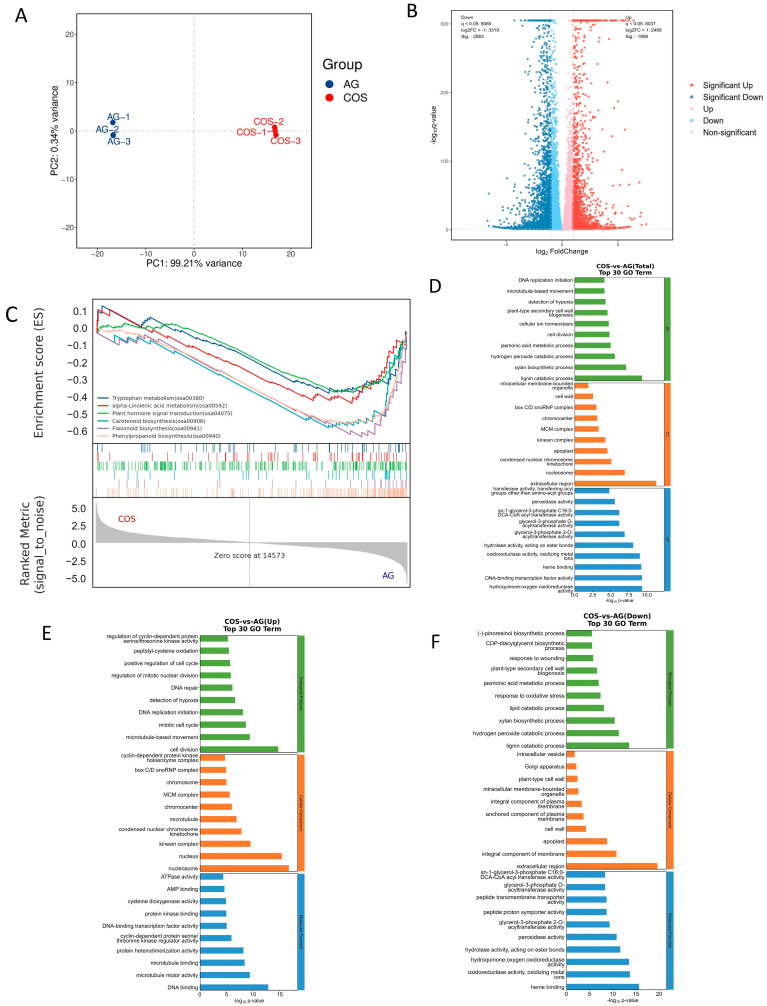
Transcriptome sequencing results. (**A**) Principal component analysis of genes. (**B**) Volcano map of differentially expressed genes. (**C**) GSEA analysis. (**D**–**F**) GO enrichment of differentially expressed genes.

A total of six samples (three from COS and three from AG) were used for the RNA-seq conducted in this study, and the quality of the sequencing met the requirements for analysis (Table S3). Through the correlation analysis between samples, it can be found that R^2^ > 0.90 under the same treatment (Figure S3). Principal component analysis (PCA) showed that there was a significant aggregation of biological duplication in the treatment (Figure 2A). The variation in PCA1 was caused by the difference in chitosan oligosaccharide treatment (99.21%), and the variation in PCA2 was caused by the individual differences between samples (0.34%). The above results show the reliability of good sample repeatability and the difference in chitosan oligosaccharides in the sample. Through the analysis of differently expressed genes, we found that there were 4472 differently expressed genes obtained by COS-vs-AG, 1908 genes were up-regulated, and 2563 genes were down-regulated (Figure 2B and Table S3).

Through GO annotation of DEGs (Figure 2D–F), the differential genes up-regulated by COS-VS-AG were mainly enriched in the detection of hypoxia (GO:0070483), peptidyl−cysteine oxidation (GO:0018171), ATPase activity (GO:0016887), and cysteine dioxygenase activity (GO:0017172). The down-regulated differential genes were mainly enriched in the jasmonic acid metabolic process (GO:0009694), response to oxidative stress (GO:0006979), hydrogen peroxide catabolic process (GO:0042744), peroxidase activity (GO:0004601), and response to wounding (GO:0009611). Comprehensive analysis of up- and down-regulated genes showed that all differential genes were mainly concentrated in the hydrogen peroxide catabolic process (GO:0042744), jasmonic acid metabolic process (GO:0009694), detection of hypoxia (GO:0018171), and peroxidase activity (GO:0004601).

Through KEGG annotation of DEGs, the differential genes up-regulated by COS-VS-AG were discovered to be mainly enriched in glycolysis/gluconeogenesis (osa00010), cysteine and methionine metabolism (osa00270), and arginine and proline metabolism (osa00330). The down-regulated differential genes were mainly enriched in phenylpropanoid biosynthesis (osa00940), plant hormone signal transduction (osa04075), flavonoid biosynthesis (osa00941), tryptophan metabolism (osa00380), alpha-linolenic acid metabolism (osa00592), and carotenoid biosynthesis (osa00906). Comprehensive analysis of up- and down-regulated genes showed that all differential genes were mainly concentrated in phenylpropanoid biosynthesis (osa00940), glycolysis/gluconeogenesis (osa00010), plant hormone signal transduction (osa04075), tryptophan metabolism (osa00380), and carotenoid biosynthesis (osa00906) (Figure S4).

Gene set enrichment analysis was performed on pathways related to antioxidant capacity, glucose metabolism, auxin synthesis and conduction, and jasmonic acid synthesis and conduction (Figure 2C). Through Gene Set Enrichment Analysis (GESA) analysis of KEGG enriched pathways, the expression levels of gene clusters in these pathways were discovered to be significantly activated under AG treatment, including phenylpropanoid biosynthesis (osa00940), flavonoid biosynthesis (osa00941), tryptophan metabolism (osa00380), alpha-linolenic acid metabolism (osa00592), plant hormone signal transduction (osa04075), and carotenoid biosynthesis (osa00906). It showed that chitosan oligosaccharide soaking inhibited the expression of related genes.

In GO enrichment (Table 1), gene clusters in glycolytic processes (GO:0006096), gluconeogenesis (GO:0006094), response to hypoxia (GO:0001666), response to reactive oxygen species (GO:0000302), and auxin polar transport (GO:0009926) were found to be significantly activated under COS treatment. It showed that exogenous chitosan oligosaccharides soaking promoted the expression of related genes. At the same time, for the response to oxidative stress (GO:0006979), the jasmonic acid metabolic process (GO:0009694), the response to abscisic acid (GO:0009737), the carotenoid biological process (GO:0016117), oxidase activity (GO:0016491), oxidase activity, oxidizing metals (GO:0016722), UDP-glucosyltransferase activity (GO:0035251), and UDP-glycosyltransferase activity (GO:0008194), the expression levels of gene clusters in these pathways were significantly activated under AG treatment. It showed that chitosan oligosaccharide soaking inhibited the expression of related genes.

### 3.4. Metabolomic Differences of Seed Soaking with Chitosan Oligosaccharides in Improving the Flooding Tolerance of Rice

**Figure 3:**
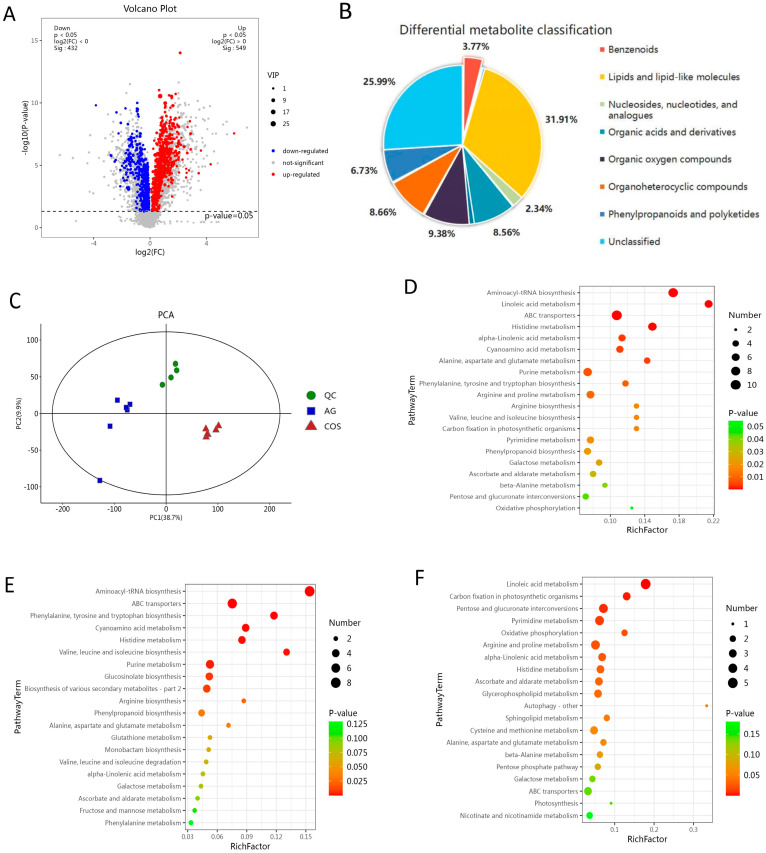
Differential metabolite analysis. (**A**) Differential metabolite volcano plot. (**B**) Differential metabolite classification. (**C**) Principal component analysis of metabolites. (**D**–**F**) KEGG enrichment of differential metabolites.

A non-targeted metabonomic analysis was performed on 6-day seedlings and found that there were 981 differential metabolites in COS-vs-AG, including 549 up-regulated and 432 down-regulated (Figure 3A). These metabolites belong to 16 categories (Table S4); in detail, lipids and lipid-like molecules took the largest proportion, with 313 differential metabolites accounting for 31.91%. Of the differential metabolites, 92 were organic oxygen compounds, accounting for 9.38%; 85 differential metabolites were organohetericyclic compounds, accounting for 8.66%; 84 differential metabolites were organic acids and derivatives, accounting for 8.56%; phenylpropanoids and polyketides made up 66 differential metabolites, accounting for 6.73%; and benzenoids made up 37 differential metabolites, accounting for 3.77% (Figure 3B). PCA analysis found that the samples were effectively separated and the QC samples had good stability, confirming that the metabolome samples can be well sequenced and analyzed (Figure 3C).

The differential metabolites were analyzed by metabolic pathway enrichment based on the KEGG database (Figure 3D–F). The up-regulated metabolites were mainly enriched in phenylalanine, tyrosine, and tryptophan biosynthesis (osa00400), arginine biosynthesis (osa00220), glutathione metabolism (osa00480), alpha-linolenic acid metabolism (osa00592), and ascorbate and aldarate metabolism (osa00053). Down-regulated metabolites were mainly enriched in linoleic acid metabolism (osa00591), oxidative phosphorylation (osa00190), arginine and proline metabolism (osa00330), alpha-linolenic acid metabolism (osa00592), and ascorbate and aldarate metabolism (osa00053). According to the comprehensive analysis, the up/down-regulated metabolites were mainly enriched in linoleic acid metabolism (osa00591), alpha-linolenic acid metabolism (osa00592), phenylalanine, tyrosine and tryptophan biosynthesis (osa00400), arginine and proline metabolism(osa00330), and ascorbate and aldarate metabolism (osa00053).

### 3.5. Combined Analysis of Transcriptome and Metabolome of Chitosan Oligosaccharides Seed Soaking’s Improvement Effect on the Flooding Tolerance of Rice Seedlings

It was discovered that the following processes, the tryptophan synthesis and metabolism–auxin signaling pathway, the jasmonic acid biosynthesis and signal transduction pathway, and the β-carotene biosynthesis–abscisic acid biosynthesis and signal transduction pathway were significantly affected by chitosan oligosaccharide seed soaking according to GO and KEGG enrichment. It is reasonable to speculate that these pathways may be the joint pathways of chitosan oligosaccharides to alleviate rice flooding stress after seed soaking.

#### 3.5.1. Tryptophan Synthesis and Metabolism–Auxin Signaling Pathway

**Figure 4:**
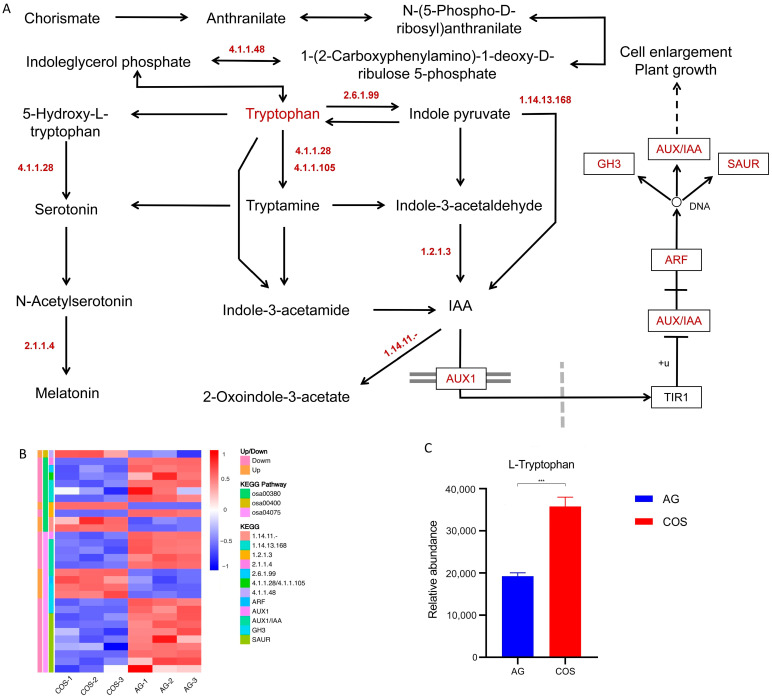
Tryptophan anabolism and auxin synthesis and signaling. (**A**) Tryptophan anabolism and auxin synthesis pathway. (**B**) Differential expression gene heatmap. (**C**) L-tryptophan relative abundance. ***: significant at the 0.001 probability level (*p* < 0.001).

In this study, L-tryptophan was identified as the differential metabolite of metabolomics analysis. The L-tryptophan content of the COS treatment was found to be significantly higher than that of the AG treatment (Figure 4C). There are many genes that are differentially expressed in this metabolic pathway (Table S5). In the process of tryptophan synthesis, the expression of *OsR498G0612846300.01* regulating indole-3-glycerol phosphate synthase (EC:4.1.1.48) was significantly up-regulated compared with the AG control COS treatment. In the process of tryptophan metabolism, the expression of *OsCOMT* regulating acetylserotonin O-methyltransferase (EC:2.1.1.4) was significantly down-regulated by COS treatment. The expression of the gene *FIB* regulating L-tryptophan-pyruvate aminotransferase (EC:2.6.1.99) was significantly down-regulated by COS treatment. The expression of the gene *OsTDC1* regulating aromatic-L-amino-acid/L-tryptophan decarboxylase (EC:4.1.1.28/4.1.1.105) was significantly down-regulated by COS treatment. The expression of *OsYUCCA2*, *OsYUCCA6*, and *OsYUCCA7* regulating indole-3-pyruvate monooxygenase (EC:1.14.13.168) was significantly down-regulated by COS treatment. The expression of *ALDH2a* regulating aldehyde dehydrogenase (EC:1.2.1.3) was significantly up-regulated in COS treatment, and the expression of related *OsALDH3E2* expression was significantly down-regulated in COS treatment. The expression of *OsR498G0204218000.01* and DAO regulating 2-oxoglutarate-dependent dioxygenase (EC:1.14.11.-) was significantly down-regulated by COS treatment.

In the process of auxin signaling, the expression of *OsAUX5* regulating AUX1 was significantly down-regulated by COS treatment. The expression of *OsR498G0100306900.01*, *OsIAA3*, *OsR498G0204677500.01*, and *OsR498G0511208200.01* regulating AUX1/IAA was significantly down-regulated by COS treatment, while the expression of *OsR498G0307212800.01* was significantly up-regulated by COS treatment. The expression of *OsARF11* regulating ARF was significantly up-regulated in COS treatment. The expression of *OsJAR2* and *OsGH3-8* regulating GH3 was significantly up-regulated in COS treatment, while the expression of *OsGH3-1* and *OsGH3-4* was significantly down-regulated in COS treatment. The expression of OsR498G0665926600.01, *OsR498G0102616400.01*, *OsR498G0409284200.01*, *OsR498G1222145600.01*, *OsR498G0203665100.01*, *OsR498G0613300200.01*, *OsR498G0613260000.01*, and *OsR498G0409249700.01* regulating SAUR was significantly down-regulated in COS treatment (Figure 4).

#### 3.5.2. Jasmonic Acid Biosynthesis and Signal Transduction

**Figure 5:**
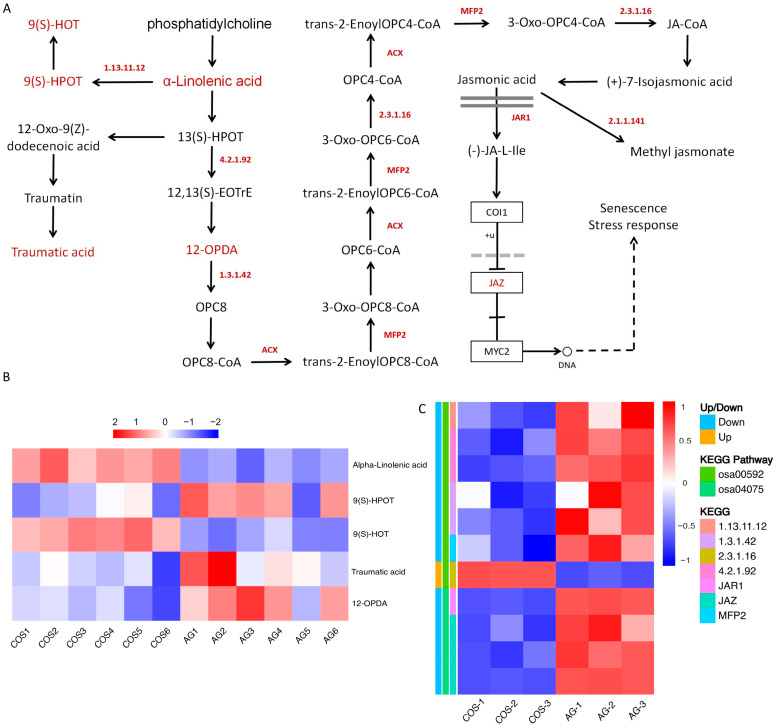
Jasmonic acid biosynthesis and signal transduction. (**A**) Jasmonic acid biosynthesis and metabolic process. (**B**) Differential metabolite relative abundance heat map. (**C**) Differential expression gene heatmap.

Through multi-omics analysis (Figure 5A), it was discovered that alpha-linolenic acid (10E, 12Z, 15Z)-(9S)-9-hydroperoxyoctadeca-10,12,15-trienoic acid (9(S)-HPOT), (9S)-(10E, 12Z, 15Z)-9-hydroxyoctadecatri-10,12,15-enoic acid (9(S)-HOT), traumatic acid, and (9S,13S,15Z)-12-oxophyto-10,15-dienoate (12-OPDA) were the main differential metabolites (Figure 5B). Among them, alpha-linolenic acid, 9(S)-HOT, and 12-OPDA metabolites increased in COS treatment compared with AG treatment, while 9(S)-HPOT and traumatic acid metabolites decreased in COS treatment compared with AG treatment.

Analysis of differential genes revealed that many key genes were affected in the process of jasmonic acid biosynthesis and signaling (Table S6). During jasmonic acid synthesis, the expression of *OsLOX1* regulating lipoxygenase (EC:1.13.11.12) was significantly down-regulated by COS treatment. The expression of genes *OsAOS3* and *OsAOS4* regulating hydroperoxide dehydratase (EC:4.2.1.92) was significantly down-regulated in COS treatment. The expression of genes *OsR498G0100971200.0* and *OsOPR1* regulating 12-oxophytodienoic acid reductase (EC:1.3.1.42) was significantly down-regulated in COS treatment. The expression of *OsR498G0509768000.01* regulating MFP2 was significantly down-regulated by COS treatment. The expression of *OsKAT1* regulating acetyl-CoA acyltransferase 1 (EC:2.3.1.16) was significantly up-regulated in COS treatment. During jasmonic acid signaling, the expression of *OsJAR1* regulating JAR was significantly down-regulated by COS treatment. The expression of genes *OsR498G0408455300.01*, *OsJAZ4*, and *OsJAZ8* regulating JAZ was significantly down-regulated by COS treatment (Figure 5).

#### 3.5.3. β-Carotene Biosynthesis–Abscisic Acid Biosynthesis and Signal Transduction

**Figure 6:**
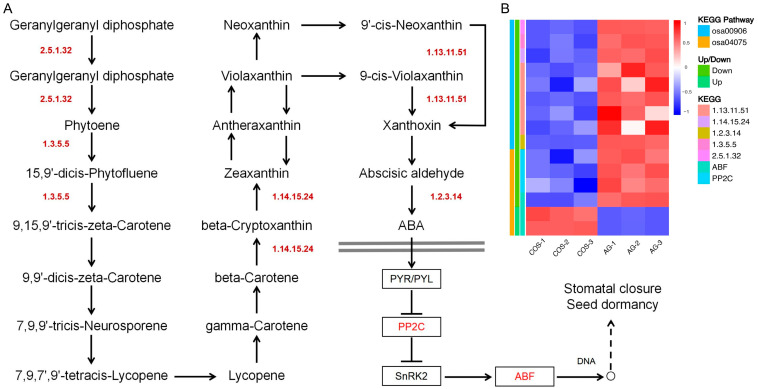
β-Carotene biosynthesis–abscisic acid biosynthesis and signal transduction. (**A**) β-Carotene biosynthesis–abscisic acid synthesis pathway. (**B**) Differential expression gene heatmap.

Analyzing the biosynthesis of β-carotene, we found the presence of many DEGs enriched in the pathway (Table S7). The expression of *OsR498G0613340000.01* controlling 15-cis-phytoene synthase (EC:2.5.1.32) was significantly down-regulated in COS treatment (Figure 6B). The expression of OsPDS controlling 15-cis-phytoene desaturase (EC:1.3.5.5) was significantly down-regulated in COS treatment. During the synthesis of abscisic acid, the expression of *OsR498G1019092700.01* controlling beta-carotene 3-hydroxylase (EC:1.14.15.24) was significantly down-regulated in COS treatment. The expression of *OsR498G0713538700.01*, *OsNCED1*, *OsNCED2*, *OsNCED3*, and *OsNCED5* controlling 9-cis-epoxycarotenoid dioxygenase (EC:1.13.11.51) was significantly down-regulated in COS treatment. The expression of *OsAO3* controlling abscisic-aldehyde oxidase (EC:1.2.3.14) was significantly down-regulated in COS treatment. In the process of abscisic acid signaling, the expression of genes *OsSIPP2C1*, *OsPP2C09*, and *OsPP2C30* controlling PP2C was significantly down-regulated by COS treatment. The expression of *OsbZIP23* controlling ABF was significantly down-regulated by COS treatment, while that of *OsbZIP09* and *OsbZIP12* was significantly up-regulated (Figure 6).

### 3.6. Field Effect Verification of the Effect of Seed Soaking with Chitosan Oligosaccharides on Improving the Submergence Tolerance of Rice

To further verify the effect of soaking seeds with chitosan oligosaccharides on improving the flooding tolerance of rice, a field validation trial was conducted. Two rice varieties, YLY948 and NX42, were selected for validation, which were suitable for cultivation in the ecological zones tested. ANOVA statistics showed that the phenotypic indicators differed significantly between varieties and treatments. There were no significant variety × treatment interaction effects on root length, number of roots, or fresh weight. By sampling seedlings 7 days after sowing, the seedling height of rice under flooding stress was significantly increased, but root length, fresh weight, and stem base width were reduced. Analysis of the number of basic seedlings showed that flooding stress seriously affected the formation of the rice population, and the number of basic seedlings for the two varieties decreased by 29.45% and 31.70%, respectively, under flooding stress. Such results indicated that the growth and development of rice were affected by a hypoxic environment. At the same time, we found that exogenous chitosan oligosaccharides affected many phenotypes of rice seedlings under flooding stress, and the seedling height of YLY948 and NX42 increased by 8.20 and 11.23%, respectively; the root length increased by 30.42 and 33.63%; the number of roots increased by 16.44 and 10.00%; the fresh weight increased by 6.61% and 1.61%; while the stem base width had no significant difference. For the number of basic seedlings, COS increased by 16.64 and 10.81% over AG (Table 2). These results indicate that chitosan oligosaccharides can significantly reduce seedling growth injury in flooded fields after sowing, stabilize the total number of seedlings in rice populations, and have applications in agricultural production.

## 4. Discussion

Previous research has shown that rice varieties with higher levels of antioxidants are more tolerant of flooding. Therefore, we theorized that without altering the genotype, the flooding tolerance of seedlings could be enhanced by applying substances that boost antioxidants [5]. The screening results showed that compounds such as chitosan oligosaccharide and melatonin, which enhance the antioxidant capacity of plants, can enhance the flood tolerance of seedlings.

Continuous flooding has a significant impact on the rice’s growth because it results in the production of a high volume of oxygen radicals within the cell, disrupting the balance of the oxygen radical scavenging mechanism [12,34]. This disturbance triggers various physiological reactions, including lipid peroxidation of the cell membrane and alterations in cell membrane permeability. The antioxidant system’s enzyme activities can indicate a plant’s ability to withstand stress. Analysis of seedlings showed that SOD, CAT, and GST enzyme activities significantly rose following chitosan oligosaccharide treatment, indicating an enhanced antioxidant system [35]. Proline and malondialdehyde can respond to the degree of cell membrane damage. In our study, we found that chitosan oligosaccharide-soaked seeds were able to alter proline and malondialdehyde content, suggesting that chitosan oligosaccharides protect cell membranes. In addition, relative conductivity measurements demonstrated that exogenous chitosan oligosaccharides improved cell membrane stability. These results show that chitosan oligosaccharide can significantly enhance the ability to remove free radicals in seedlings experiencing flooding stress, leading to improved stability of cell membrane permeability and ensuring normal cell function [36,37]. This effect may be attributed to the fact that the increase in the deacetylation of chitosan oligosaccharide led to an increase in the number of free amino groups, resulting in a higher number of free amino groups. These amino groups can then react with unpaired electrons to inhibit the formation of free radicals [38]. Previous studies have proposed that alpha-amylase activity during germination is an important physiological marker for assessing tolerance to flooding [39,40]. Our research also observed that the addition of chitosan oligosaccharide alleviated the decrease in alpha-amylase activity due to flooding stress, which accounts for the sustained growth of seedlings.

Many studies have shown that different environmental factors such as high temperature, low temperature, drought, and salt stress affect the synthesis and distribution of endogenous hormones in plants, thus causing changes in plant physiology and biochemistry [41]. Numerous studies have been conducted to show that ethylene, GA, IAA, JA, and ABA are regulated by different mechanisms under flooding stress in rice [12]. This study found that these phytohormones were closely related to the soaking of seeds in chitosan oligosaccharides. Tryptophan is able to synthesize melatonin and IAA, which are related to growth and development [42]. In our study, we found that the soaking of seeds in chitosan oligosaccharides resulted in an increase in tryptophan content. The transcriptome sequencing results showed that exogenous chitosan oligosaccharides negatively regulated the genes responsible for melatonin synthesis. It may be that the antioxidant function of exogenous chitosan oligosaccharides replaces the efficacy of endogenous melatonin [43]. During IAA synthesis and signaling, we found that genes that have been reported to be up-regulated under abiotic stress (e.g., *YUCCA*, *ALDH*, *GH3*, *AUX*, etc.) were negatively regulated by chitosan oligosaccharides in this study [44,45,46]. It is possible that the antioxidant capacity of chitosan oligosaccharides itself alleviated the intensity of flooding stress, thereby leading to the down-regulation of the expression of the genes involved, which in turn allowed for the sustained synthesis of IAA to maintain seedling growth.

JA and ABA are important phytohormones that play key roles in plant resistance to stressful environments. Research has demonstrated that PYL4, ABI1, and OST1, important proteins of abscisic acid, interact with JAZ1 proteins, and these results suggest that the synergistic regulation of JA and ABA is an important way for plants to adapt to the stress environment for growth [47]. The transcriptome analysis showed that chitosan oligosaccharides negatively regulated the important genes involved in the response to stress, such as *OsAOS4*, *JAR1*, *JAZ*, and *PP2*, associated with the JA and ABA pathways [48,49,50,51]. Chitosan oligosaccharides induced JA accumulation in rice, and JA plays a signaling role in chitosan oligosaccharides-mediated plant defense responses [52]. As a result, we theorized that soaking seeds in chitosan oligosaccharides alleviated the mechanism of JA and ABA’s regulation of adversity by alleviating the intensity of flooding stress. In addition, the metabolite traumatic acid, which is associated with plant damage, was found to be down-regulated in the chitosan oligosaccharides-treated group, which also demonstrated that chitosan seed dipping could alleviate the damage suffered by rice under flooding stress.

## 5. Conclusions

**Figure 7:**
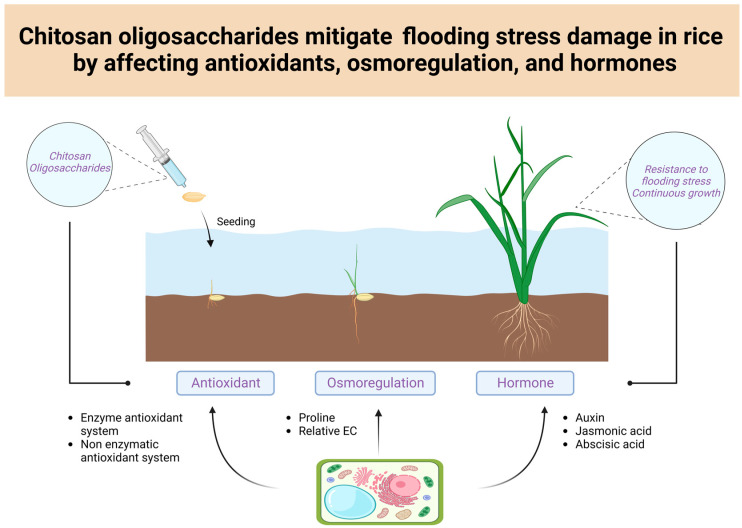
Mechanism of action of soaking seeds in chitosan oligosaccharides to alleviate flooding stress.

This study reveals that chitosan oligosaccharides could alleviate the inhibition of rice growth under flooding stress to a certain degree, ensure the development of cells and tissues by increasing the antioxidant capacity and stabilizing the cell membrane permeability of rice, and regulate the growth and development of rice seedlings under flooding stress by regulating IAA, JA, and ABA (Figure 7). Similarly, field trials have demonstrated the practical effectiveness of chitosan in alleviating flooding stress in agricultural production, which has great application value.

## Figures and Tables

**Table 1:** GSEA of GO enrichment.

Functional Category	Term	ES	NES	*p*-Value	FDR
GO-BP	glycolytic process (GO:0006096)	0.50	2.06	0.00	0.00
	gluconeogenesis (GO:0006094)	0.64	1.99	0.00	0.00
	response to hypoxia (GO:0001666)	0.58	1.91	0.00	0.00
	response to oxidative stress (GO:0006979)	−0.48	−2.18	0.00	0.00
	jasmonic acid metabolic process (GO:0009694)	−0.72	−2.24	0.00	0.00
	response to abscisic acid (GO:0009737)	−0.30	−1.39	0.00	0.17
	response to reactive oxygen species (GO:0000302)	0.42	1.55	0.02	0.08
	auxin polar transport (GO:0009926)	0.38	1.46	0.02	0.12
	carotenoid biosynthetic process (GO:0016117)	−0.47	−1.47	0.04	0.13
GO-MF	oxidoreductase activity (GO:0016491)	−0.38	−1.72	0.00	0.02
	methyl indole-3-acetate esterase activity (GO:0080030)	−0.71	−1.98	0.00	0.00
	peroxidase activity (GO:0004601)	−0.55	−2.39	0.00	0.00
	oxidoreductase activity, oxidizing metal ions (GO:0016722)	−0.73	−2.44	0.00	0.00
	UDP-glucosyltransferase activity (GO:0035251)	−0.52	−1.76	0.00	0.01
	UDP-glycosyltransferase activity (GO:0008194)	−0.39	−1.53	0.02	0.06

GO-BP: gene ontology—biological process, GO-MF: gene ontology—molecular function, ES: enrichment score, NES: normalized enrichment score, FDR: false discovery rate.

**Table 2:** Field phenotypic results of different rice varieties.

Variety	Treatment	Seedling Height (cm)	Root Length (cm)	Number of Roots	Stem Base Width (mm)	Fresh Weight (g)	Basic Number of Seedlings
YLY948	AG	4.70 ± 0.25 b	2.95 ± 0.49 b	3.20 ± 0.22 b	16.40 ± 0.11 b	0.62 ± 0.01 b	22.33 ± 0.48 c
	COS	5.12 ± 0.13 a	4.24 ± 0.31 a	3.83 ± 0.35 a	16.27 ± 0.18 b	0.66 ± 0.01 a	26.79 ± 0.43 b
	CK	4.07 ± 0.07 c	4.83 ± 0.40 a	3.93 ± 0.43 a	16.72 ± 0.07 a	0.65 ± 0.01 a	31.65 ± 1.51 a
NX42	AG	3.24 ± 0.16 b	2.27 ± 0.19 c	2.0 ± 0.29 b	14.71 ± 0.45 b	0.61 ± 0.02 a	15.92 ± 1.07 c
	COS	3.65 ± 0.19 a	3.42 ± 0.53 b	2.2 ± 0.08 ab	15.11 ± 0.21 b	0.62 ± 0.02 a	17.85 ± 0.31 b
	CK	3.03 ± 0.12 b	3.97 ± 0.15 a	2.4 ± 0.18 a	16.66 ± 0.08 a	0.63 ± 0.01 a	23.31 ± 1.32 a
Variety (V)		**	**	**	**	**	**
Treatment (T)		**	**	**	**	**	**
V × T		*	ns	ns	**	ns	*

Columns with the same letter are not significantly different at *p* < 0.05; one-way ANOVA, followed by Tukey’s honestly significantly different tests. ns: not significant. *: Significant at the 0.05 probability level. **: Significant at the 0.01 probability level.

## Data Availability

Data is contained within the article.

## Supplementary Materials

The following supporting information can be downloaded at: https://www.mdpi.com/article/10.3390/antiox13050521/s1.
