# Supplementary figures and images for: Chitosan Oligosaccharides Mitigate Flooding Stress Damage in Rice by Affecting Antioxidants, Osmoregulation, and Hormones
Source: Antioxidants (Basel). 2024 Apr 26;13(5):521. doi: 10.3390/antiox13050521 (PMC11117766; doi:10.3390/antiox13050521)

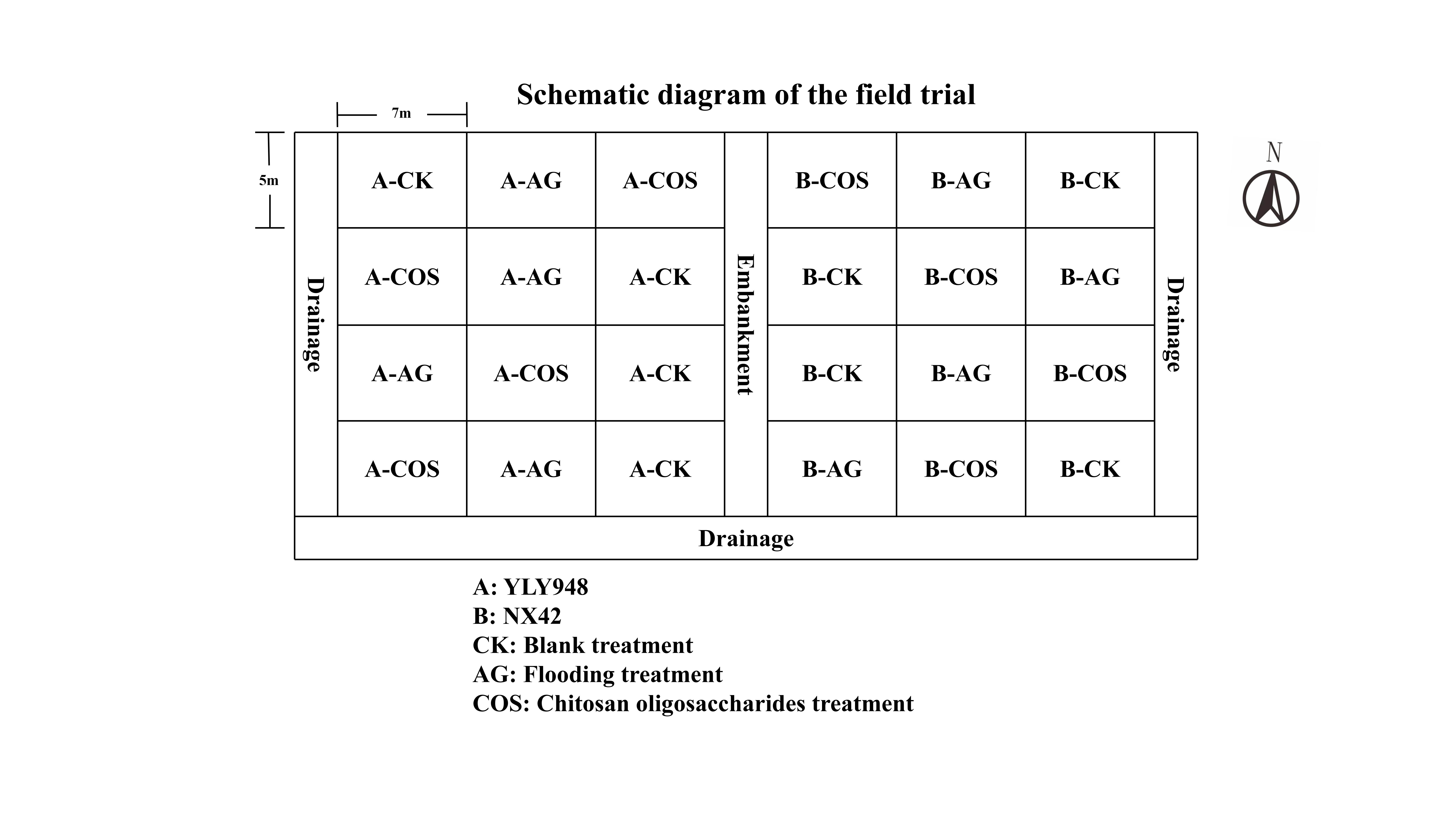

Supplement: Supplementary file 1 [file antioxidants-13-00521-s001.zip › Fig. S1.jpg]

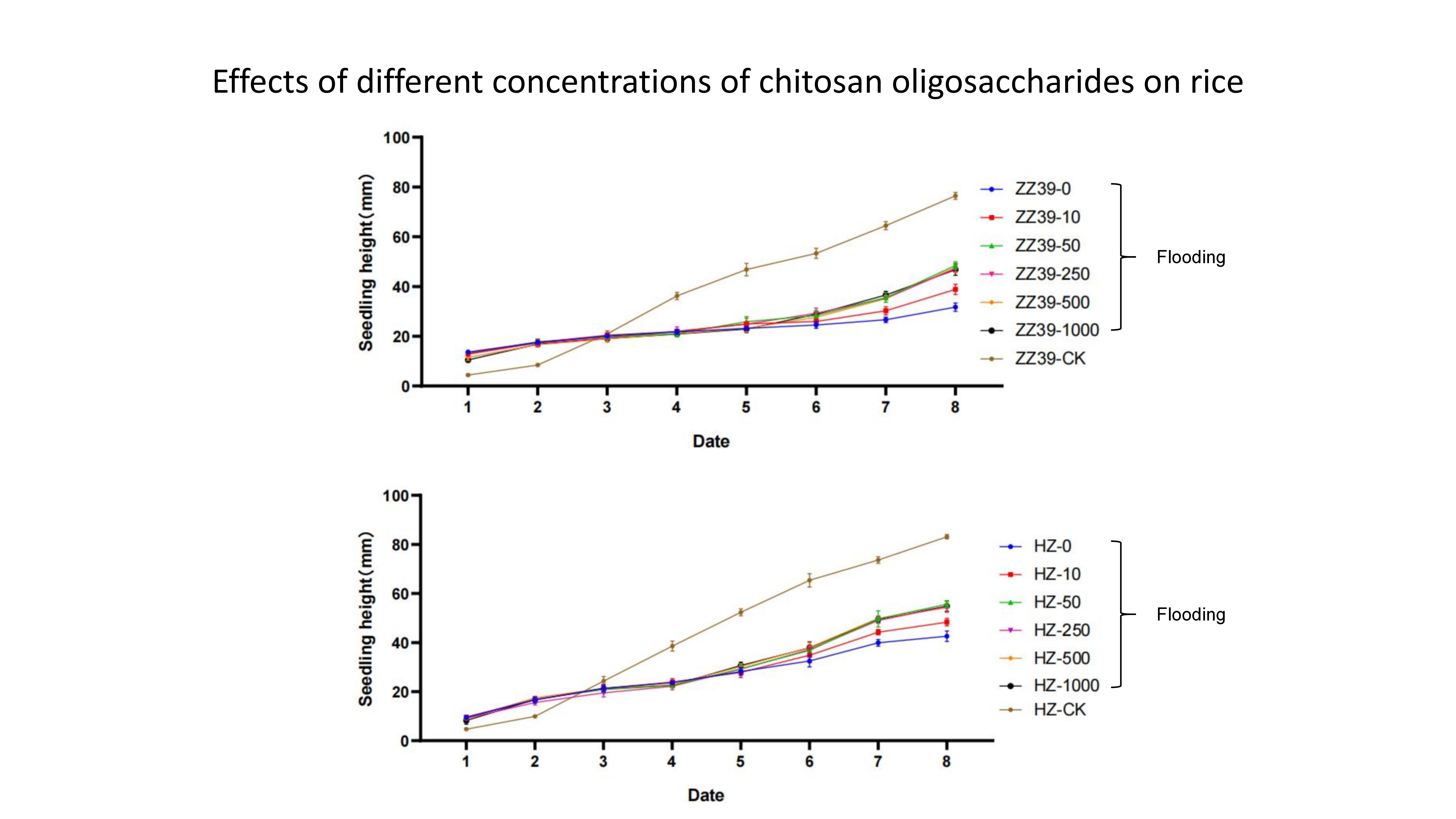

Supplement: Supplementary file 1 [file antioxidants-13-00521-s001.zip › Fig. S2-new.jpg]

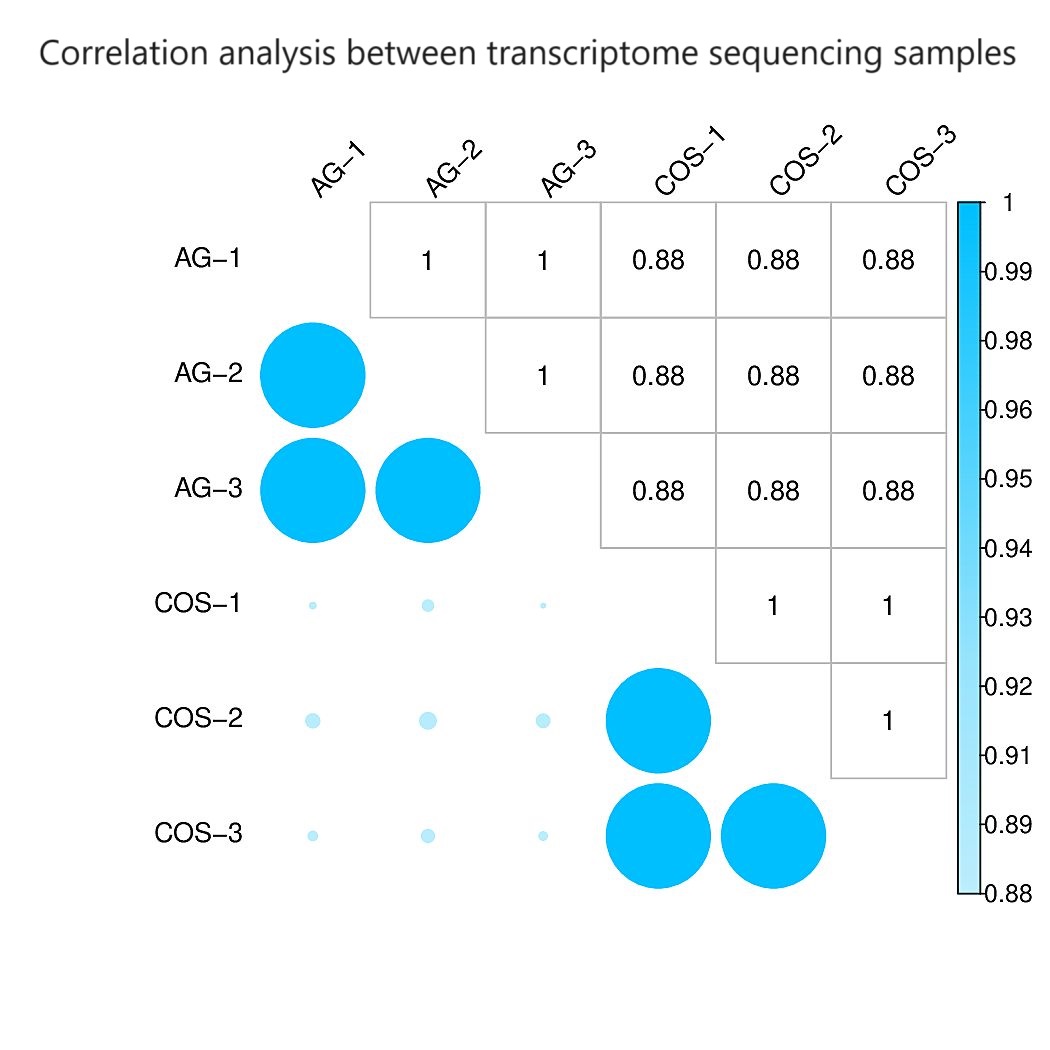

Supplement: Supplementary file 1 [file antioxidants-13-00521-s001.zip › Fig. S3.jpg]

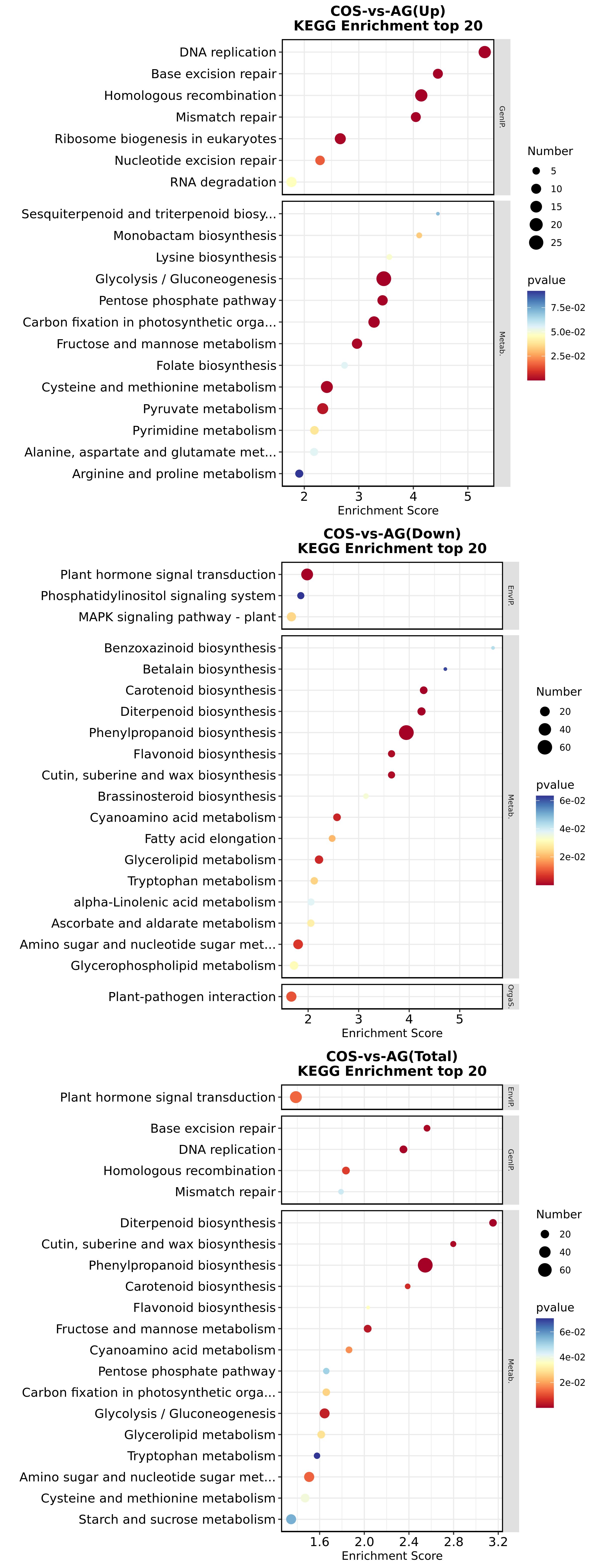

Supplement: Supplementary file 1 [file antioxidants-13-00521-s001.zip › Fig. S4.jpg]
